# Effect of Mercury in Acute Rheumatism

**Published:** 1811-10

**Authors:** D. H. Davies

**Affiliations:** 27, Carburton-Street, Fitzroy-Square


*To the Editors of the Medical and Physical Journal.*

GENTLEMEN,

SINCE the case of Acute Rheumatism inserted in your last Number, I have met with an occurrence in my practice which I think may in some measure tend to elucidate and confirm the observations there suggested, and which may likewise serve to point out several useful hints, not so much to medical men, as to the unprofessional public.

A short time since I was requested to visit Mr. C. a young man about the age of 28. He had for years been a sufferer from disease, but was particularly subject to attacks of inflammatory Rheumatism; even in the periods of comparative health, he was never entirely free from this troublesome complaint; he always felt wandering pains about him, and on the least occasion of cold he generally experienced a very severe attack. Wearied with his uncomfortable and wretched kind of life, and having tried the prescriptions of regular and empirical practice, without any real benefit or amendment, he in a manner became desperate, and at the instance of a friend, who had been ordered by a medical man to rub in mercury for a complaint, the symptoms of which he conceived exactly resembled his, he commenced rubbing in a quantity of *ung. hyd. fort.* of the size of a *large nutmeg* every night and morning, a quantity which I conceive to be about ʒij, or nearly so, of the ointment. He continued persevering in this plan for about four or five days without any very visible effect, except a slight affection of the mouth; but on the seventh day from the commencement of his using the ointment, the most violent effects were produced, apparently of a sudden. Very copious spitting came on, the breath became extremely fœtid, the mouth and lips were swelled to a very great extent, the eyes had a sunk, hollow appearance; a compleat hoarseness, so that the voice was scarcely distinguishable, occurred; the throat was extremely sore; large evacuations of blood were frequently made from the mouth, and the whole countenance assumed a hideous appearance. This was the period when I first saw him. I ought to have previously observed, he commenced using the mercury when he was labouring under a very severe attack of rheumatism, and though previously tormented with pain, he never experienced a single return of it after the mercury had begun to affect his mouth. When I saw him he was excessively *reduced and debilitated*, not only from the immediate effect of the mineral, but, if I am allowed to use the the term, from a mechanical cause, he being incapacitated from taking nourishment almost of any description from the state of his mouth; I therefore requested the attendants to give him as much nourishment as possible in the form of broth, soup, &c. directed him to take the decoction of bark with tincture, and to gargle the mouth frequently. Perseverance in this plan, with attention to keeping the bowels perfectly open, gradually produced convalescence, and he is now perfectly recovered. Since the period of the mercury affecting his constitution, he has not felt the least return of rheumatic pains. He is now gone to Margate in order to bathe, where I hear he continues perfectly free from any vestige of his former complaint. Time will determine, but I firmly believe he is *permanently cured*, through the influence of a most violent yet useful medicine. The conclusions that may be drawn from this case are very evident. First, it is clear that the mercury cured the disease by inducing a different action in the system, and suspending or rather eradicating the previously morbid one: Secondly, the random and unwarrantable way in which the application was used, and the unpleasant consequences that followed, ought to serve as a useful lesson to the unprofessional part of the public not to tamper with important medicines, as the consequences attendant when made use of in improper doses, and in diseases that do not require their use, is often dangerous, and likewise that their exhibition ought to be always superintended by a medical man. Thirdly, I think it is in this case clearly evident, that a slight but at the same time a satisfactory affection of the mouth, answers every purpose, in inflammatory diseases, of a *perfect ptyalism*; as it is to be observed, that every trace of the disease vanishes upon the appearance of this criterion of the medicine having affected the constitution. Sincerely hoping the above remarks may prove useful, I leave them to the consideration of your readers.

Your obliged,

D. H. DAVIES.

*27, Carburton-Street, Fitzroy-Square.*

